# On the Importance of Processing Conditions for the Nutritional Characteristics of Homogenized Composite Meals Intended for Infants

**DOI:** 10.3390/nu8060340

**Published:** 2016-06-03

**Authors:** Elin Östman, Anna Forslund, Eden Tareke, Inger Björck

**Affiliations:** Food for Health Science Centre, Lund University, P.O. Box 124, 221 00 Lund, Sweden; anna.k.forslund@gmail.com (A.F.); eden.tareke@food-health-science.lu.se (E.T.); inger.bjorck@food-health-science.lu.se (I.B.)

**Keywords:** infant food, glycemia, insulinemia, human, advanced glycation end products, carboxymethyl-lysine, early protein hypothesis, protein quality, carbohydrate digestibility, glycemic index

## Abstract

The nutritional quality of infant food is an important consideration in the effort to prevent a further increase in the rate of childhood obesity. We hypothesized that the canning of composite infant meals would lead to elevated contents of carboxymethyl-lysine (CML) and favor high glycemic and insulinemic responses compared with milder heat treatment conditions. We have compared composite infant pasta Bolognese meals that were either conventionally canned (CANPBol), or prepared by microwave cooking (MWPBol). A meal where the pasta and Bolognese sauce were separate during microwave cooking (MWP_CANBol) was also included. The infant meals were tested at breakfast in healthy adults using white wheat bread (WWB) as reference. A standardized lunch meal was served at 240 min and blood was collected from fasting to 360 min after breakfast. The 2-h glucose response (iAUC) was lower following the test meals than with WWB. The insulin response was lower after the MWP_CANBol (−47%, *p* = 0.0000) but markedly higher after CANPBol (+40%, *p* = 0.0019), compared with WWB. A combined measure of the glucose and insulin responses (ISI_composite_) revealed that MWP_CANBol resulted in 94% better insulin sensitivity than CANPBol. Additionally, the separate processing of the meal components in MWP_CANBol resulted in 39% lower CML levels than the CANPBol. It was therefore concluded that intake of commercially canned composite infant meals leads to reduced postprandial insulin sensitivity and increased exposure to oxidative stress promoting agents.

## 1. Introduction

In the development of infant formulas, weaning food, and composite infant meals, the main aim has traditionally been to ensure the provision of adequate amounts of essential nutrients. Great interest has therefore been devoted to the availability of certain nutrients, such as protein and selected minerals. Two nutritional quality characteristics that are emphasized in food for adults are the availability of carbohydrates affecting the glycemic response, and the presence of process-induced advanced glycation end products (AGEs), which affect the biological value of the protein. Carbohydrate-rich foods with low glycemic impact were recently classified as relevant for the prevention and treatment of type 2 diabetes, coronary heart disease, and probably obesity [1]. High-glycemic meals have been associated with the increased activation of inflammatory markers in the postprandial phase [2]. Elevated intakes of AGEs are of interest due to their association with cardiometabolic risk markers and pathological conditions such as diabetes [3,4,5]. In the case of powder-based weaning foods, considerable and varying amounts of AGEs were recently reported in milk powder, infant formulas, and gruel [6,7]. Furthermore, high intakes of ultra-processed food products were recently positively and independently associated with increased prevalence of excess weight gain and obesity in different age groups in Brazil [8]. It is interesting to note that composite canned meals intended for small children are subjected to high-temperature treatment. Excessive heat treatment is known to affect the availability of the carbohydrate component [9], and the homogenization of composite infant meals prior to canning is likely to further increase the availability of the carbohydrate components for digestion and absorption [10]. Additionally, homogenization of the meal components could be expected to boost AGE formation by enhancing the accessibility of the necessary precursors.

Based on the above, we hypothesized that the canning of homogenized composite infant meals may render carbohydrates and protein rapidly available for digestion and absorption, leading to high blood glucose and insulin responses. Furthermore, it was hypothesized that the increased availability of carbohydrates and proteins would result in the formation of higher levels of AGEs than in less harshly processed composite meals. The objectives of the present work were to establish some of the nutritional quality characteristics of canned homogenized composite meals intended for infants, and to compare them with more gently processed alternatives.

## 2. Methods

Two studies were performed, the first of which involved a commercially canned pasta Bolognese meal and a canned composite meal with beef and white beans (Study 1). In the second study, a commercially canned pasta Bolognese meal (purée with some intact soft pieces) was again used and compared with a similar but microwave heat-treated meal (cracked spaghetti with minced meat and vegetables). Separately boiled cracked spaghetti served with a commercially canned meat sauce was also included. White wheat bread (WWB) was used as a reference [11] in both studies to allow the determination of the glycemic index (GI) and the insulinemic index (II). The GI is defined as the incremental area under the blood glucose curve (iAUC) following the intake of a test meal, expressed as a percentage of an equi-carbohydrate reference meal, eaten by the same subject. The II is calculated similarly, based on the corresponding insulin responses. WWB is a starch-rich product considered to be a more physiologically relevant reference product than pure glucose, due to its more complex food matrix. The meal studies were performed in healthy adults to investigate the postprandial effects on glucose and insulin. In Study 2, non-esterified fatty acids (NEFAs) and triglycerides (TGs) were also analyzed. *N*-carboxymethyl-lysine (CML) was used as a marker of AGEs. The CML contents in some commercially canned composite infant meals and relevant composite frozen meals intended for adults were included for comparison. All test products were heated to eating temperature prior to the analysis of CML, determination of rate of *in vitro* starch hydrolysis, or being served as a test meal.

### 2.1. General Study Design

Healthy adult volunteers were recruited for the studies. All test subjects gave their informed consent, and were aware that they could withdraw from the study at any time. The studies were approved by the Regional Ethical Review Board in Lund (LU 558-01 and 2012/615). The test meals and the reference meal were served as breakfast in a random order, after overnight fasting. The tests were performed approximately one week apart and commenced at the same time in the morning. Subjects were instructed to maintain their regular lifestyle throughout the period of the study. The day prior to a test they were told to avoid alcohol, excessive physical activity, and food rich in dietary fiber. On the evening (21.00–22.00) before each test, the subjects were instructed to eat a standardized meal consisting of white wheat bread with spread and drink of their own choice. However, the subjects were instructed to have the same evening meal before each test.

### 2.2. Study 1

Five men and four women aged 24–41 years, with normal body mass indices (23.1 ± 2.7 kg/m^2^; mean ± SD), normal fasting blood glucose (4.4 ± 0.05 mmol/L; mean ± SEM), and who were not taking any medication, participated in the study. The test subjects were recruited and the study performed between October 1999 and February 2000. Test subjects were recruited by advertising on notice boards around the Lund University (LU) campus, and by contacting former volunteers. Two commercially available canned composite meals, “canned Meat&Pasta” and “canned Meat&Beans”, intended for infants aged 12 months, were studied. The meals were microwave heated according to the manufacturer’s instructions before serving. Both test meals and the reference WWB meal contained 30 g of potentially available carbohydrates [12]. Both the test meals and the reference meal were served with 250 mL water and followed by 150 mL coffee or tea. Finger-prick capillary blood samples were taken repeatedly up to 120 min after ingesting the meal for the analysis of blood glucose concentrations (glucose oxidase-peroxidase reagent) and serum insulin (Insulin ELISA, Mercodia AB, Uppsala, Sweden).

#### Product Characterization

Levels of CML were determined in freeze-dried samples of selected canned composite infant meals and corresponding frozen ready-to-eat meals intended for adults using gas chromatography mass spectrometry (GC-MS) according to Birlouez-Aragon [3]. The results are presented in Table 1. The *in vitro* rate of starch hydrolysis (hydrolysis index, HI) was analyzed for the meals using the method described by Granfeldt *et al.* [13]. Before HI-analysis, samples of the composite meals were rinsed in a strainer using tap water to obtain the intact pieces of the carbohydrate sources (beans and pasta).

### 2.3. Study 2

#### 2.3.1. Recipes and Processing Conditions

Canned pasta Bolognese (CANPBol) for infants aged 12 months was bought in the local supermarket, and two other test meals were prepared in the laboratory. In order to investigate the possible advantages of minimal processing of a composite meal, an in-pack pasteurization method was used (MicVac AB, Mölndal, Sweden). The microwave cooked test meal (MWPBol) was based on a recipe resembling that of the CANPBol meal, and contained: 60 g water, 50 g crushed tomatoes (ICA, Solna, Sweden), 17 g carrots, 17 g yellow onions, 16 g minced beef, 13 g manually cracked spaghetti (ICA Italia, Solna, Sweden), 10 g tomato purée (ICA), 10 g celery root, 2 g corn starch (Maizena, Unilever, Solna, Sweden), 2 g rapeseed oil (ICA), 0.15 g iodized salt (Falksalt, Ab Hanson & Möhring, Halmstad, Sweden), 0.2 g dry oregano (Santa Maria, Mölndal, Sweden), 0.1 g dry basil (Santa Maria), and 0.07 g white pepper (Santa Maria). A test meal consisting of separately microwave-cooked pieces of spaghetti (ICA Italia) and canned Bolognese sauce (Felix Köttfärsås Original, Orkla Foods, Eslöv, Sweden) was used to study the effect of preparing the meal components separately (MWP_CANBol). All pasta and canned Bolognese sauce were bought from one batch. In the case of the MWPBol meal, all dried/canned ingredients and minced beef was bought from one batch, but vegetables were bought fresh every week. The minced beef was frozen in portions and thawed prior to the preparation of each serving. Each MWPBol portion was prepared the evening before the test by combining the minced meat with small pieces of the vegetables, crushed tomatoes, spices, and cracked spaghetti. After sealing the tray with plastic film, the portion was cooked in a microwave oven for 8 min, chilled on ice and then stored overnight in a refrigerator. Just before serving, MWPBol meal was reheated in microwave oven for 1.5 min. In the case of the MWP_CANBol meal, cracked spaghetti was boiled in water for 7.5 min using a similar tray sealed with plastic film in microwave oven and stored in the refrigerator overnight. In the morning, the pasta was heated separately for 2 min, and the canned Bolognese sauce was heated separately for 2.5 min, in the microwave oven. The pasta and Bolognese sauce were then mixed on the plate before serving. Each CANPBol portion was weighed on a plate just before serving, and microwave heated for 2.5 min, with breaks for stirring after 1 and 2 min.

#### 2.3.2. Product Characterization

All test and reference meals in Study 2 were standardized so as to contain 35 g available starch, according to Holm *et al.* [12]. The CANPBol meal and the canned Bolognese sauce were analyzed as bought, without preparation. The microwave-cooked spaghetti was re-heated before analysis, and the starch content converted to dry matter basis. The protein contents in the test meals were determined using an elemental analyzer (FlashEA 1112, Thermo Fischer Scientific Inc., Waltham, MA, USA). The fat contents in the commercial products were estimated from the manufacturers’ declarations. In the case of WWB, estimates of the protein and fat contents were based on a previous analysis of a similar product [14]. CML was determined using high-pressure liquid chromatography mass spectrometry (HPLC-MS/MS), with an Accela UHPLC pump with an autoinjector coupled to an LTQ VelosPro Orbitrap mass spectrometer (Thermo Scientific, Waltham, MA, USA). The MS/MS was run in positive electrospray ionization ion trap mode, detecting two selected reaction monitoring (SRM) transitions for CML and two for the internal standard. Xcalibur software (ver. 2.2, Thermo Scientific) was used for both data acquisition and evaluation. Samples were prepared by hydrolyzing 0.3 g sample for 12 h at 110 °C, using 6 M HCl, together with isotope-labelled d4-CML (Larodan Fine Chemicals AB, Malmö, Sweden) as internal standard. CML was extracted after hydrolysis using solid phase extraction (Telos*neo*PCX, Teknolab Sorbent AB, Västra Frölunda, Sweden). All the dried samples were reconstituted in 0.01% (v/v) nonafluoropentanoic acid (Sigma-Aldrich, Steinheim, Germany) and centrifuged before analysis. Solid phase extraction, chromatographic parameters, ion source parameters, and the SRM transitions were the same as described by Tareke *et al.* [6,7]. CML analyses were performed on three different days. To check any instrumental inconsistency, duplicates of each sample were analyzed on one occasion. The portion sizes, macronutrient and energy compositions, and the CML contents are presented in Table 2.

#### 2.3.3. Subjects

The inclusion criteria were being a healthy, non-smoker with normal weight (body mass index, BMI, 19–25 kg/m^2^), aged 18–40 years, with a stable body weight over the previous two months. Exclusion criteria were being a vegetarian/vegan and/or having food allergies, lactose intolerance, having any disease or taking any medication that might affect the study, as well as being pregnant or lactating.

#### 2.3.4. Sample Collection and Protocol

The subjects arrived at the laboratory at 7:45 a.m. after overnight fasting. A peripheral venous catheter (BD Venflon Dickinson, Helsingborg, Sweden) was inserted into an antecubital vein. Capillary plasma glucose and venous blood samples were collected, after which the individually assigned test meal was served, together with 250 g of tap water (time 0). All meals were tolerated and finished within 10–15 min, except for one test person who needed 20 min to finish eating the CANPBol meal. Blood samples were taken at 15, 30, 45, 60, 90, 120, 240, 270, 300, 330, and 360 min after the beginning of the meal. One hundred and thirty-five minutes after the meal, each subject was given 200 g water to be drunk within 15 min. Two hundred and forty-five minutes after the start of the meal, a standardized lunch, consisting of a ready-to-eat dish of 400 g (2320 kJ) pasta with tomatoes, basil, and mozzarella cheese (Pasta al Pomodoro, Gooh, Lantmännen, Sweden) was served. The lunch meal was served with 250 g tap water, and was to be consumed within 20 min.

#### 2.3.5. Blood Analysis

Blood glucose was analyzed in capillary blood (HemoCue^®^B-glucose, HemoCue AB, Ängelholm, Sweden). Serum was obtained from venous blood collected in CAT tubes (BD Vacutainer, ref. 368492) and left to clot for 60 min before being centrifuged at 3500 rpm for 10 min at 4 °C. Plasma was obtained in EDTA tubes (BD Vacutainer, ref 368274) and put on ice for a maximum of 30 min until centrifuged at 3500 rpm for 10 min at 4 °C. Samples were then aliquoted in Eppendorf tubes and stored immediately at −20 °C until analysis. Serum insulin and serum TGs were analyzed by a lab at Skåne University Hospital (Clinical Chemistry, Region Skåne, Sweden), whereas NEFAs were analyzed at the department using a colorimetric assay (NEFA C, ACS-ACOD method, WAKO Chemicals GmbH, Neuss, Germany).

## 3. Statistical Calculations

The least number of subjects required to detect a difference in the GI with a power of 80% at a level of *p* < 0.05, is ten [15]. The sample size in Study 1 was nine, and in order to improve the power of Study 2, twenty-one subjects were included. The iAUCs for glucose and insulin were calculated using GraphPad Prism (GraphPad Software, San Diego, CA, USA) and the trapezoid model. All areas below the baseline were excluded from the calculations. The results are expressed as means ± SEM. Differences resulting in *p* < 0.05 were considered statistically significant.

### 3.1. Study 1

The 120 min blood glucose and insulin iAUCs were used to determine GI and II, respectively. The statistical differences were evaluated using the general linear model (ANOVA) followed by Tukey’s multiple comparisons test using Minitab software (ver. 13, Minitab Inc., State College, PA, USA). 

### 3.2. Study 2

Differences in CML content between the test meals and the reference meal were evaluated with one-way ANOVA using GraphPad Prism (as described above). The composite insulin sensitivity index (ISI_composite_), also called the Matsuda index, was calculated for both the test meals and the reference meal in order to assess the insulin sensitivity. 

ISI_composite_ = 10,000/√[fasting glucose (mmol/L) × fasting insulin (nmol/L) × glucose iAUC 0–120 min (mmol·min/L) × insulin iAUC 0–120 min (nmol·min/L)] [16,17]. 

The effects of the test meals and reference meal on glucose and insulin responses, as well as on the NEFA and TG levels, were evaluated using the PROC MIXED SAS procedure. The subject was treated as a random effect and the test meal as a fixed effect. The fixed effect of corresponding baseline (fasting) values was included as a covariate, and time × meal interactions were tested. All models were tested for the normality of residuals. To adjust for multiple comparisons of significant effects, the Tukey–Kramer *post hoc* significance test was performed. Statistical analyses of metabolic outcomes were performed using SAS 9.2 (SAS Institute Inc., Cary, NC, USA) and Minitab software (ver. 16). The data are presented as iAUC values or least-square means (LSMs) ± SEM. The data were also tested for outliers. The fasting level of insulin was found to be an outlier for one subject, and the result was therefore not included in the insulin analyses. In addition, insulin data were lacking for one subject 30 min after the CANPBol meal and for another 240 min after the MWP_CANBol meal. Due to the missing values, those two test meals were excluded from the calculations for insulin. Another subject missed the CANPBol meal, resulting in *n* = 20 for the CANPBol meal for all blood tests except insulin, where *n* = 18.

## 4. Results

### 4.1. Study 1

The canned meals intended for infants contained three to four times higher levels of CML (expressed as mg CML/g protein) than frozen meals with similar ingredients but intended for adults (Table 1). The HI for canned beans was 44, which was significantly lower (*p* < 0.05) than the HI of both the canned pasta (109) and WWB reference (100).

The glycemic response to canned Meat&Beans (GI = 48 ± 11) was significantly lower (*p* < 0.05) than that after the reference WWB meal (GI = 100). The GI of canned Meat&Pasta (79 ± 12) did not differ from either of the other two meals. No significant differences were found in the insulin responses, and the II values were 100, 91 ± 13, and 140 ± 30 for WWB, canned Meat&Beans, and canned Meat&Pasta, respectively.

### 4.2. Study 2

Twenty-one healthy volunteers (14 men and 7 women) participated in Study 2. All female subjects were taking birth control medication. The mean BMI was 21.8 ± 0.3 kg/m^2^ and mean age 24.3 ± 0.9 years (±SEM). All subjects had normal fasting blood glucose levels (5.4 ± 0.06 mmol/L). The recruitment of test subjects and the study were performed from November 2012 to March 2013. Recruitment was performed by advertising on notice boards at and around the LU campus as well as by contacting former volunteers. The macronutrient composition and CML content of the various foods are presented in Table 2. All test and reference meals were similar in content of available carbohydrates and the protein and fat levels as well as energy contents were within the same range for all composite test meals. CANPBol and MWPBol contained significantly higher levels of CML compared with MWP_CANBol. The CML-content of WWB was significantly lower than in all test meals.

#### 4.2.1. Blood Glucose Responses

**Figure 1:**
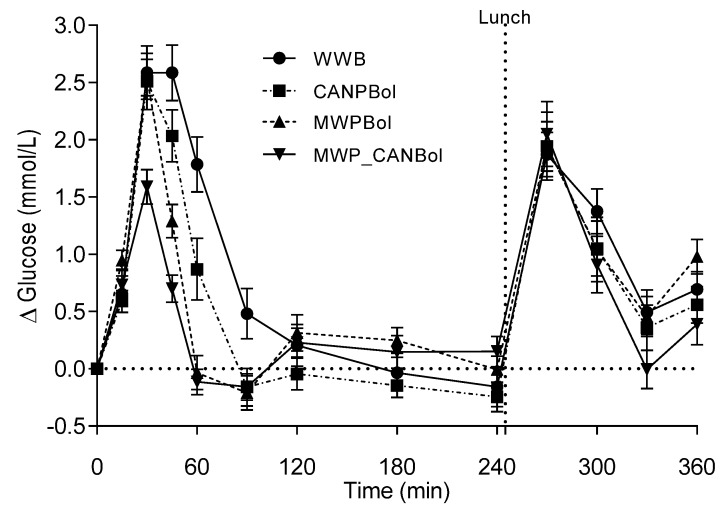
Mean (±SEM) changes in glucose response after the test meals and reference meal and the subsequent standardized lunch (Study 2).

No differences were observed in fasting glucose levels. There was a significant time × meal interaction (*p* < 0.0001) over the 360 min follow-up (Figure 1). Incremental glucose responses (0–120 min) were significantly lower (31%–63%) following all the test meals than after the WWB meal (Table 3). Furthermore, the glucose response (iAUC and GI) after the MWP_CANBol meal was significantly lower than those after the two other test meals, during the same time period. After the standardized lunch meal (iAUC 240–360) the glucose response following the MWP_CANBol breakfast was significantly lower compared to WWB and CANPBol. The cumulative glucose response over the entire test period (iAUC 0–360 min) showed that overall glycemia following the MWPBol and MWP_CANBol meals was lower than following the CANPBol meal and WWB reference meal.

#### 4.2.2. Insulin Response

**Figure 2:**
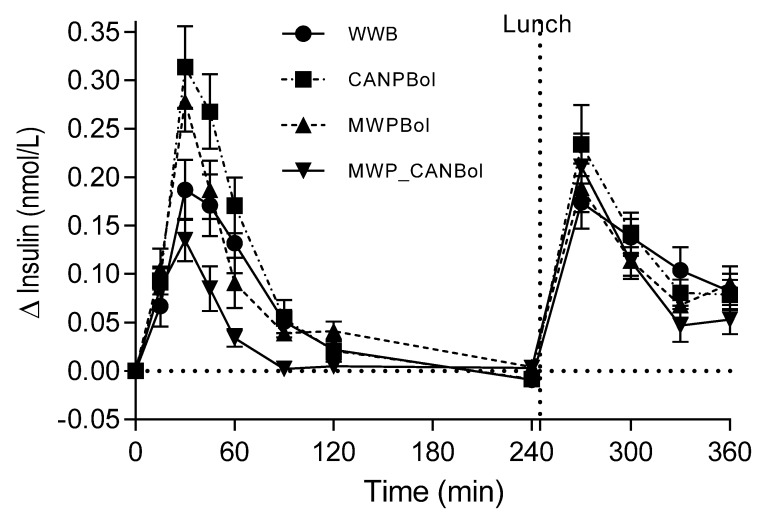
Mean (±SEM) changes in insulin response after the intake of the test meals and reference meal, and subsequent standardized lunch (Study 2).

No differences were observed in fasting insulin levels. There was no significant time × meal interaction over the 360 min study period (Figure 2). During the first 45 min, the insulin level was significantly higher following the CANPBol meal (59%) and the MWPBol meal (40%) than the MWP_CANBol meal and the WWB meal. The incremental insulin responses during the periods 0–120 and 0–360 min were significantly lower after the MWP_CANBol meal than after all other meals. In addition, the insulin response during 0–120 min was significantly higher following the CANPBol meal than the WWB reference meal.

#### 4.2.3. Blood Lipids

**Figure 3:**
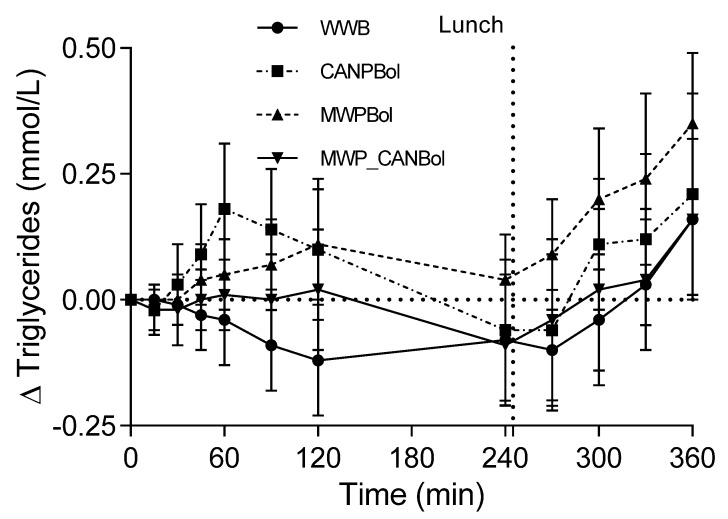
Mean (±SEM) changes in triglyceride response after the intake of the test meals and reference meal, and a subsequent standardized lunch (Study 2).

No differences were observed in fasting levels of NEFAs or TGs and no significant time × meal interactions were observed for either of the lipid variables (*p* < 0.0001). Pairwise comparisons of NEFA levels at 240 min showed that they were significantly lower after the MWPBol and MWP_CANBol meals than after the WWB meal (*p* = 0.0392 and 0.0001, respectively, Figure S1). The overall TG response was significantly higher after the CANPBol (*p* = 0.0216) and MWPBol (*p* = 0.0008) meals than after the WWB meal (Figure 3). Furthermore, the level of TGs was significantly higher following the MWPBol meal than after the MWP_CANBol meal (*p* = 0.0041) over the 360 min.

## 5. Discussion

The major finding of this study is that canned composite pasta Bolognese meals, processed according to a common procedure for infant meals, elicited substantially higher postprandial metabolic responses in healthy adults than meals intended for adults prepared with conventional cooking conditions. In fact, the infant CANPBol meal elicited an almost two-fold higher postprandial glucose and a 2.6 times higher insulin level during the first 2 h, compared with a meal of canned meat sauce served with gently and separately cooked pasta (MWP_CANBol). When combining the glucose and insulin data in a composite index, ISI_composite_, the insulin sensitivity was improved by 94% after the MWP_CANBol meal, compared with the CANPBol meal. The significantly higher insulin response (+59%) during the first 45 min after the CANPBol meal, compared to the WWB meal, cannot be explained by the corresponding increase in glucose, since this was reduced by 10% (not statistically significant). Instead, we suggest that a fraction of the proteins was rendered highly soluble by the canning process, and may thus exert insulinotrophic effects. Surprisingly, the microwave-cooked composite meal (MWPBol) also led to a significant increase in early insulin response, but no significant differences were found in insulin, compared with WWB, over 120 min. The separately processed meal, MWP_CANBol, resulted in substantially lower glucose (−63%) and insulin (−47%) responses, compared with WWB. Apparently, the canning of Bolognese sauce *per se* did not result in an elevated insulin response, and the reduction in both glucose and insulin is in line with previous findings regarding pasta meals, where a low glucose peak and a late net increment with an accompanying lowering of insulin responses have been reported [18]. Holm *et al.* have previously reported a higher postprandial glycemic response in healthy adults to canned pasta, compared to cooked pasta [9]. They observed glucose responses that were twice as high after canned then after boiled spaghetti, and the insulin response was also significantly increased by canning. A suggested mechanism for the rapid uptake of carbohydrates following the intake of canned pasta was the excessive swelling of the starch, resulting in a very soft and easily digestible texture. The significantly reduced glucose response following the canned Meat&Beans in Study 1 is probably the result of some remaining intact cell structures following homogenization and canning of the composite bean meal [19]. This was also illustrated by the lower *in vitro* rate of starch hydrolysis for the bean component in the canned Meat&Beans meal (HI = 44), than in the canned Meat&Pasta meal (HI = 109) and the WWB reference meal (HI = 100). It should be noted though, that only the intact beans were included in HI-analysis, since they stayed in the strainer after washing. The high HI found for canned pasta is in line with the findings of a previous study indicating that homogenized spaghetti (cooked spaghetti treated for 35 s in a food processor) had a higher GI (73) than intact spaghetti (60), but was still lower than that for bread baked using spaghetti ingredients (100) [18]. Taken together, these findings indicate that canning of pasta disrupts the texture.

The substantial reduction in postprandial glycemic response after the MWP_CANBol meal, compared to the other meals, is further reflected by the improved glucose tolerance following a standardized lunch, manifested by a 38% lower glucose response, than after the WWB breakfast. Another indication of improved insulin sensitivity at the time of lunch (240 min) following the MWP_CANBol breakfast was the reduced NEFA levels. This is in agreement with other reports showing that suppressed NEFA levels between meals improves glucose tolerance following a second meal [20]. The increase in TG levels tended to be higher after the intake of CANPBol than after the other meals over the first postprandial hour. This may be related to a higher availability of lipids from this particular canned composite meal.

It has been suggested that the mechanism behind the increased risk of obesity associated with high protein intakes during infancy may be the protein-associated stimulation of insulin and IGF-1 release [21]. The results of the present study show that insulin stimulation by infant food may be related not only to the quantity of protein, but possibly also to process-induced changes in protein availability. In line with this, hydrolyzed protein appears to stimulate insulin release to a higher extent than intact protein [22].

The CML content in the MWP_CANBol meal was significantly lower than in the MWPBol meal (−34%) and the CANPBol meal (−39%). Gentle processing of the composite meal, as in the case of the MWPBol meal, was, thus, not as effective as expected in terms of reducing CML contents and no significant difference was found between the CML levels in the MWPBol and CANPBol meals. These results indicate that it may be more important to separate the protein- and carbohydrate-rich meal components during processing, than to use gentle heat treatment of a composite meal. The CML comparison in Study 1 indicates that freezing may be an interesting way forward to allow for both gentle and separate heat treatment of the different meal components. It is important to note that the formation of CML renders lysine less available. The fact that lysine is an essential amino acid is one reason to counteract CML formation during processing of all foods, and especially in products intended for infants. Additionally, dietary exposure to CML has repeatedly been linked to impaired metabolism. Consequently, a cross-over study in healthy subjects has shown that exposure to CML-levels of 5.4 mg CML/day (GC-MS analysis) for one month resulted in lower insulin sensitivity, lower plasma levels of omega-3 fatty acids, and higher concentrations of blood lipids than after a low-AGE diet (2.2 mg CML/day) [3]. Similarly, the consumption of a high-AGE diet (24.6 mg CML/day, LC-MS analysis) for one month led to increased fasting insulin and insulin resistance (HOMA-IR), compared to a diet low in AGEs (10.7 mg CML/day), in overweight women [23]. The CML content in one portion of CANPBol was 9.7 mg (LC-MS analysis), and a single meal of canned infant food would thus provide 0.75 mg CML/kg body weight in 12-month-old infants (assuming a body weight of 13 kg). This is more than twice the amount of CML ingested in the case of the high-CML diet previously mentioned where the intake in adults corresponded to 0.35 mg/kg body weight per day [23]. A recent review of randomized controlled trials has shown that high AGE intakes are associated with increased levels of TNF-alpha, which is an established biomarker of inflammation in healthy humans [24]. Furthermore, high postprandial glycemia *per se*, as seen in the current study in the case of CANPBol, has been associated with low-grade inflammation [2,25]. In summary, the intake of canned infant meals appears to result in reduced postprandial insulin sensitivity and high intakes of AGE (measured as CML). The latter is due to favorable conditions for the Maillard reaction, which may result in lower lysine availability and pro-inflammatory responses. 

It should be noted that the sample size in Study 1 was lower than that recommended for GI determinations, which may partly explain the larger variation in insulin response after the canned Meat&Pasta meal than after the CANPBol meal. It cannot be excluded that the inclusion of coffee/tea at both test and reference breakfasts in Study 1 may have influenced the postprandial insulin sensitivity and it was therefore removed from the study design in Study 2. Although the metabolic responses to the infant meals were measured in healthy adults, it is reasonable to assume that the differences seen, depending on the type of processing, would be similar in infants.

## 6. Conclusions

The findings of this study indicate an urgent need for more research on the metabolic effects of food products intended for infants and young children. The importance of considering both the quantity and quality of macronutrients when designing diets for infants was also recently pointed out by Alvisi *et al.* [26]. Gentle and separate processing of meal components seems to have a beneficial impact on human metabolism compared to canning or microwave heated mixed composite meals. With respect to CML contents, conventional cooking and freezing of meal components appears to be more beneficial in comparison with canning.

## Figures and Tables

**Table 1:** *N*-carboxymethyl-lysine (CML) content in canned and homogenized meals intended for infants and in frozen ready-to-eat meals intended for adults.

Meal	CML (mg/g Protein)
Beef stew “Kalops”	
*Canned, homogenized*	0.33
*Frozen*	0.08
Hash “Pytt i panna”	
*Canned, homogenized*	0.45
*Frozen*	0.14

Results are presented as the mean of two duplicates.

**Table 2:** Portion sizes and contents of macronutrients, energy, and CML in the test meals and reference meal in Study 2.

Meal	Portion g	Starch g/Port	Protein g/Port	Fat ^1^ g/Port	Energy kJ/Port	CML ^2,3^ mg/Port
WWB	83	35	4.9	0.7	704	0.41 ± 0.04 ^a^
CANPBol	467	34	13	11.7	1232	9.75 ± 0.89 ^b^
MWPBol	479	36	15	11.5	1292	8.98 ± 0.13 ^b^
MWP_CANBol of which:	309	35	12	9.3	1143	5.94 ± 0.64 ^c^
Canned Bolognese sauce	130	3.3	7.3	8.9	-	5.94 ± 0.64 ^c^
Pasta, uncooked	42	30	4.4	0.4	-	n.a.
Maize starch (Maizena)	2	1.7	-	-	-	n.a.

^1^ Estimated from a previous study (WWB) and manufacturers’ declarations (commercial products/ingredients). WWB, white wheat bread; CANPBol, canned mixed meal; MWP_CANBol, canned Bolognese sauce served with microwave-cooked spaghetti; MWPBol, microwave-cooked mixed meal; n.a., not analyzed; ^2^ Values are presented as mean ± SEM, *n* = 4; ^3^ Values within the row not sharing a superscript letter are significantly different, *p* < 0.05.

**Table 3:** Glucose and insulin responses ^1^, together with a measure of insulin sensitivity after the test meals and the reference meal (Study 2).

	WWB ^2^	CANPBol ^2^	Δ %	MWPBol ^2^	Δ %	MWP_CANBol ^2^	Δ %
Glucose							
GI 0–120 min	100 ^a^	79 ± 13 ^b^	−21	61 ± 5.4 ^b^	−39	38 ± 3.6 ^c^	−62
iAUC 0–45 min	68.4 ± 6.13 ^a^	61.8 ± 5.30 ^a^	−10	62.0 ± 3.40 ^a^	−9	40.6 ± 4.31 ^b^	−41
iAUC 0–120	153 ± 16.0 ^a^	104 ± 13.7 ^b^	−31	84.6 ± 10.3 ^b^	−45	57.4 ± 7.7 ^c^	−63
iAUC 240–360	148 ± 18.5 ^a^	136 ± 16.3 ^a^	−8	120 ± 12.4 ^a,b^	−19	92.2 ± 13.1 ^b^	−38
iAUC 0–360	312 ± 30.8 ^a^	240 ± 31.9 ^a,b^	−23	245 ± 37.9 ^b^	−22	206 ± 33.3 ^b^	−34
Insulin							
II 0–120 min	100 ^a^	161 ± 19 ^b^	61	129 ± 15 ^a,b^	29	57 ± 5.5 ^c^	−43
iAUC 0–45 min	5.11 ± 0.79 ^a^	8.10 ± 1.02 ^b^	59	7.15 ± 0.88 ^b^	40	4.01 ± 0.63 ^a^	−22
iAUC 0–120	11.3 ± 1.79 ^a^	15.8 ± 2.45 ^b^	40	12.4 ± 1.90 ^a,b^	10	5.95 ± 0.97 ^c^	−47
iAUC 0–360	26.4 ± 3.74 ^a^	32.2 ± 4.86 ^a^	22	26.5 ± 3.81 ^a^	0.2	20.2 ± 2.75 ^b^	−24
ISI_composite_	703 ± 82 ^a^	733 ± 98 ^a^	4	865 ± 72 ^a^	23	1421 ± 135 ^b^	102

^1^ Values are presented as mean ± SEM; ^2^ Values within the same row not sharing superscript letters are significantly different, *p* < 0.05. GI, glycemic index; iAUC, incremental area under the curve; II, insulin index; ISI_composite_, insulin sensitivity index; WWB, white wheat bread; CANPBol, canned mixed meal; MWP_CANBol, canned Bolognese sauce served with microwave-cooked spaghetti; MWPBol, microwave-cooked mixed meal.

## Supplementary Materials

The following are available online at http://www.mdpi.com/2072-6643/8/6/340/s1, Figure S1: Mean (±SEM) changes in NEFA response after the intake of the test meals and the reference meal (Study 2).
