# Supplementary material for: On the Importance of Processing Conditions for the Nutritional Characteristics of Homogenized Composite Meals Intended for Infants
Source: Nutrients. 2016 Jun 3;8(6):340. doi: 10.3390/nu8060340 (PMC4924181; doi:10.3390/nu8060340)
Supplement: Supplementary file 1 [file nutrients-08-00340-s001.docx]

**Supplementary Materials: On the Importance of Processing Conditions for the Nutritional Characteristics of Homogenized Composite Meals Intended for Infants**

Elin Östman, Anna Forslund, Eden Tareke and Inger Björck

**Figure S1.** Mean (±SEM) changes in non-esterified fatty acids (NEFA) response after the intake of the test meals and the reference meal (Study 2).
